# Robotic-assisted percutaneous coronary intervention: experience in Switzerland

**DOI:** 10.3389/fcvm.2023.1294930

**Published:** 2023-12-04

**Authors:** Jonas D. Häner, Lorenz Räber, Christina Moro, Sylvain Losdat, Stephan Windecker

**Affiliations:** ^1^ Department of Cardiology, Bern University Hospital, University of Bern, Bern, Switzerland; ^2^ CTU Bern, University of Bern, Bern, Switzerland

**Keywords:** robotic-assisted percutaneous coronary intervention, procedural success, manual assistance, manual conversion, Swiss experience

## Abstract

**Aims of the study:**

Percutaneous coronary intervention (PCI) exposes operators to ionizing radiation. Robotic-assisted PCI (RA-PCI) is a novel technology that enables interventional cardiologists to operate coronary devices remotely from a radiation-shed cockpit. The aim of this study is to describe the experience and challenges during the initiation of a RA-PCI program and to report outcomes of the first 21 patients undergoing RA-PCI in Switzerland.

**Methods:**

All patients undergoing RA-PCI using the CorPath GRX Vascular Robotic System between 06/2021 and 12/2021 at Inselspital, Bern University Hospital were included in this retrospective registry study. Baseline, procedural and clinical follow-up data were prospectively assessed as part of the Cardiobase Bern PCI registry (NCT02241291). The two endpoints of interest were clinical success [defined as <30% residual diameter stenosis in the absence of in-hospital major adverse cardiovascular events (MACE: composite of death, periprocedural myocardial infarction, target-vessel revascularization, and stroke)] and robotic success (defined as clinical success and completion of RA-PCI without or with partial manual assistance). Additional outcome measures include clinical long-term outcomes at one year.

**Results:**

Twenty-five lesions in 21 patients were treated with RA-PCI (age 62.4 ± 9.1 years, 24% female). Clinical success was achieved in 100%, and robotic success in 81% (17/21 procedures, including 4 procedures requiring partial manual assistance). Manual conversion (e.g. manual completion of the procedure) occurred in 19% (4 procedures). Reasons for manual assistance or conversion were poor guiding-catheter back-up or platform limitations (4), adverse events (2x transient slow-flow that was solved manually), safety decision (1x vasovagal reaction not related to robotic approach), and software error (1). No in-hospital MACE occurred. During 12 months of follow-up, one patient suffered a non-target-vessel myocardial infarction requiring repeat PCI.

**Conclusions:**

RA-PCI can safely be performed without clinically relevant robot-associated complications in selected patients with approximately 80% of procedures conducted without or with partial manual assistance.

## Introduction

Percutaneous coronary intervention (PCI) is the most frequently performed myocardial revascularization procedure. The intervention uses fluoroscopy and cine angiography, exposing the operator to ionizing radiation and necessitating the wear of protective lead aprons. While robotic systems became widely used in urologic, gynecologic, thoracic and abdominal surgery in the last decade (1, 2), robotic-assisted PCI (RA-PCI) is still in its infancy. The robotic system CorPath GRX allows the operator to perform the procedure remotely circumventing the use of lead aprons with the associated orthopedic injuries as well as complete protection from ionizing radiation (3). Furthermore, robotic systems promise higher interventional precision (than manual operation with visual estimation) (4). The PRECISE (5) and CORA-PCI (6) studies (for the older robot version CorPath 200) and the San Diego (7) and Ahmedabad registries (8) (for the CorPath GRX) reported clinical success similar to manual intervention with markedly reduced radiation exposure to the operator, without increase in radiation exposure to the patient. In a propensity score matched analysis even lower radiation exposure to the patient was observed (8). As such, RA-PCI is a novel technology that should be explored regarding its suitability for use in routine clinical PCI practice.

The aim of this study is to describe the experience and challenges during implementation of RA-PCI in Bern, Switzerland and to assess the safety and feasibility of the first 21 patients undergoing RA-PCI.

## Material and methods

### Study population and data source

All consecutive patients undergoing RA-PCI between 06/2021 and 12/2021 at Bern University Hospital, Switzerland, were enrolled in this cohort study with prospective data collection. Only patients with known coronary anatomy were considered for RA-PCI (e.g., staged PCI or deferred index PCI) in the early phase of the robotic program implementation. Baseline, procedural characteristics and clinical outcome data were prospectively collected in the Cardiobase Bern PCI registry (NCT02241291). Patient consent to participate in the registry was retrieved according to local regulations. The registry study was approved by the local ethics committee on human research (KEK 137/14) and was conducted in accordance with the Declaration of Helsinki. During the routine pre-interventional information, aspects of robotic PCI were explained to patients eligible for RA-PCI by the operator. An RA-PCI-specific informed consent was obtained from all patients.

### Robotic-assisted intervention

**Figure 1:**
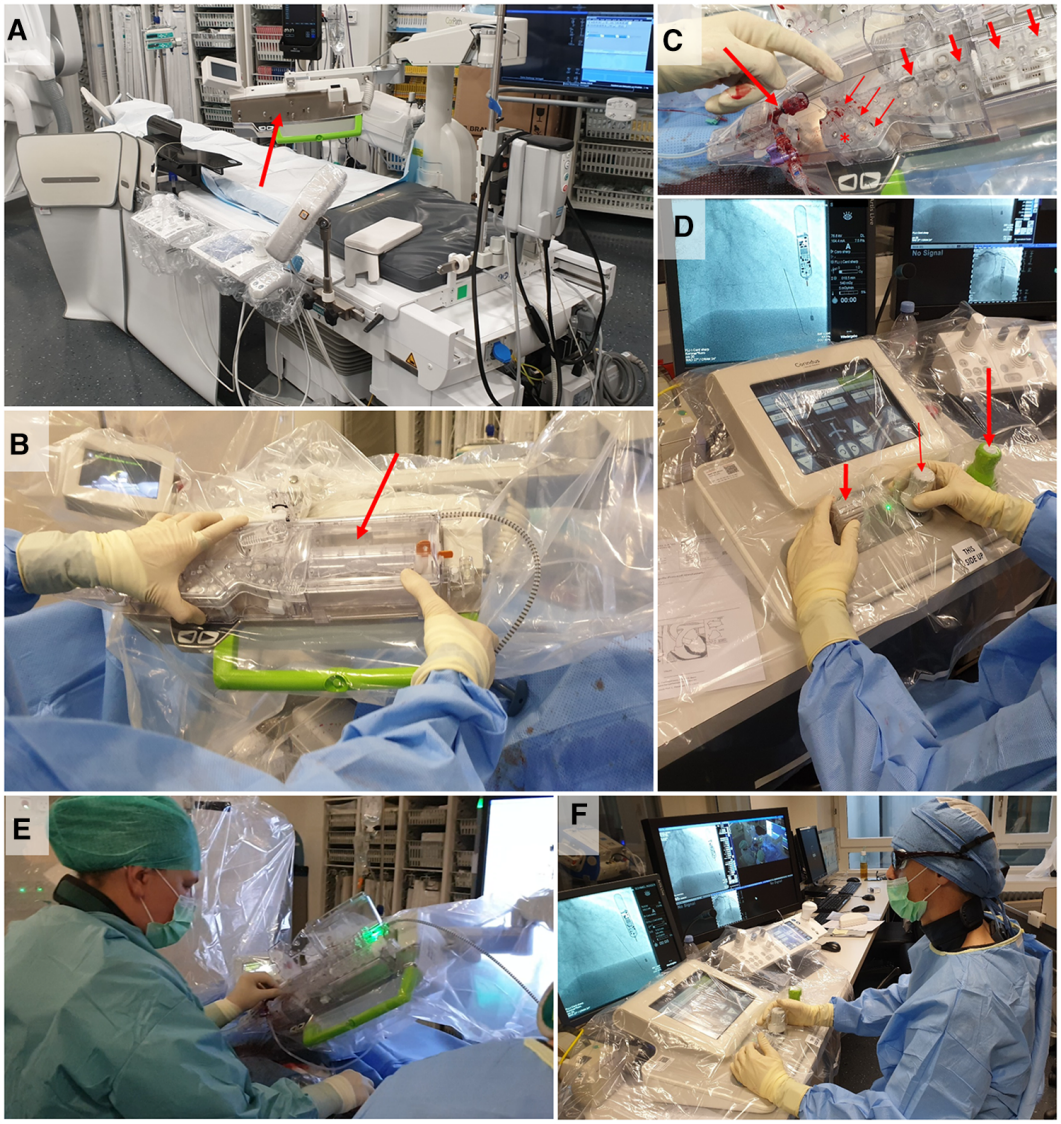
Set-up of robotic-assisted PCI. The robotic system consists of a robotic arm installed at the catheterization table (arrow in panel **A**) and an interventional cockpit in the radiation-shed control room. For robotic-assisted percutaneous coronary intervention (RA-PCI), a single-use cassette is placed on the drive platform of the robotic arm (arrow in panel **B**). After manual engagement of the coronary artery ostium with the guiding catheter, the Y-connector (large arrow in panel **C**) is inserted into the cassette. Guidewires and coronary rapid exchange devices (e.g. balloons, coronary stents) are placed in their respective lanes [panel C: short thick arrows show wire-lane; thin arrows show device-lane; the star (*) indicates the “parking” position for wires and devices]. The cockpit (panel **D**) consists of the joysticks to remotely control guiding catheter (large arrow), wire (thin arrow), and device (short thick arrow), a touch-screen to command stepwise submillimeter movements, and a screen displaying live fluoroscopy / angiography images. The console can be used with or without a sterile cover, depending on whether or not the operator is wearing sterile clothing. During RA-PCI, the robotic drive platform is handled (e.g. loading / exchanging devices) by the specially trained Catheterization Laboratory technician (panel **E**), while the operator supervises and remotely conducts the procedure from the cockpit (panel **F**).

The CorPath GRX robotic platform (Corindus, a Siemens Healthineers Company, Waltham, MA, USA) was used for remote-controlled RA-PCI. The set-up is illustrated in Figure 1. The system consists of a robotic arm fixed to the catheterization table and of a radiation-shed interventional cockpit. Prior to connecting the robotic system, a standard guiding catheter (deployed either by radial or femoral access) was used to manually intubate the ostium of the target coronary artery. A single-use cassette was then placed on the robotic drive platform of the robotic arm and was manually connected to the guiding-catheter. A specially trained Catheterization Laboratory technician introduced the guidewire, and, subsequently rapid-exchange ballons and/or stents into the cassette. Later on, this tableside assistant was also responsible to remove the devices from the cassette again. The robotic arm was electronically connected to the interventional cockpit, which was operated by the interventional cardiologist using remote-controlled joysticks. It allowed the operator to manipulate the guiding catheter (rotate, advance, and retract), the guidewire (rotate, advance and retract), and the balloon or stent (advance and retract) (9). Of note, PCI or diagnostic techniques other than balloon/stent delivery or intracoronary imaging cannot be applied with the current CorPath GRX robotic platform. By manual switching of guidewires and/or devices from the “parking” position to the drive lane by the Catheterization Laboratory technician, the operator was enabled to control different guidewires/devices sequentially. Live fluoroscopic images and hemodynamic data were displayed to the operator on cockpit monitors. Contrast administration and balloon/stent inflations were done by the Catheterization Laboratory technician based on the operator's instruction. At the end of the procedure the robotic system was manually disconnected from the guiding catheter and the guiding catheter was manually removed.

During the intervention, patients received weight-adjusted heparin to achieve an activated clotting time >250 s. Antithrombotic therapy after PCI was prescribed according to institutional guideline recommendations.

### Study outcomes and endpoint definitions

The two endpoints of interest were rates of clinical success and robotic success. ***Clinical success*** was defined as the achievement of <30% residual diameter stenosis by visual estimation and absence of in-hospital major adverse cardiovascular events (MACE), a composite of death, myocardial infarction [according to the Society for Cardiovascular Angiography and Interventions definition (10)], clinically-driven target vessel revascularization, and stroke (6, 11, 12).

***Robotic success*** was defined as clinical success and completion of the RA-PCI without or with partial manual assistance (6, 11). We also recorded reasons of partial manual assistance and manual conversion. Partial manual assistance was defined as temporary disconnection of the robotic system to enable bedside manipulation of the guiding catheter, guidewire, monorail-device and/or additional devices not compatible with the robotic platform (e.g., microcatheters, optical coherence tomography), with completion of the procedure using the re-connected robotic drive. Manual conversion was defined as disconnection of the robotic drive with manual completion of the intervention.

Success rates of single robotic steps (e.g., guidewire delivery, device delivery, device retrieval) were also recorded. These definitions and results are provided in the Appendix.

Total procedural time was defined as the time from manual intubation of the coronary artery ostium to the removal of the guiding catheter, whereas the robotic time was defined as the time from connection of the guiding catheter to the robotic drive to removal of the guiding catheter independent of whether the procedure was completed robotically or whether a manual conversion had occurred.

Clinical outcomes were assessed throughout 1 year of follow-up and included target lesion failure (composite of cardiac death, target-vessel myocardial infarction, and clinically driven target lesion revascularization), any death, any myocardial infarction and any clinically driven revascularization.

### Statistical analysis

Only descriptive statistics were performed. Since this is a retrospective cohort study including all patients undergoing RA-PCI between the initiation of the RA-PCI program in June 2021 and December 2021, no formal sample size calculation was conducted. Baseline clinical and procedural characteristics as well as procedural and clinical outcome results are presented as counts and percentages for categorical variables and as mean ± standard deviation or median [interquartile range] for continuous variables. Statistics were performed using Stata 17.

## Results

### Study population and procedural characteristics

Between June 2021 and December 2021, RA-PCI was performed in 21 patients with 25 lesion. All robotic procedures were performed in the setting of planned staged PCIs. Baseline and procedural characteristics are summarized in Tables 1, 2.

**Table 1:** Baseline characteristics.

	*N* = 21 patients/procedures
Clinical parameters	
Age, years	62.4 ± 9.1
Female	5 (24%)
Body-mass index, kg/m^2^	29.1 ± 3.8
Arterial hypertension	12 (57%)
Diabetes mellitus	5 (24%)
Dyslipidemia	9 (43%)
Current or history of smoking	12 (57%)
Prior percutaneous coronary intervention	21 (100%)
Prior coronary artery bypass graft surgery	0 (0%)
History of myocardial infarction	15 (71%)
History of stroke	1 (5%)
Peripheral arterial disease	0 (0%)
Clinical presentation	
Chronic coronary syndrome	21 (100%)
Acute coronary syndrome	0 (0%)
Left-ventricular ejection fraction, %	55% ± 9%
Laboratory parameters	
eGFR, ml/min	101 [34]
Creatine kinase, U/L	123 [146]
TnT-hs, µmol/L	12 [15]

Values are mean ± SD, median [interquartile range], or count (%). eGFR, estimated glomerular filtration rate using Cockcroft-Gault; TnT-hs, high-sensitivity Troponin T.

**Table 2:** Procedural characteristics.

Procedural characteristics on procedure level (*N* = 21 patients/procedures)	
Access site	
Radial, right	19 (90%)
Radial, left	1 (5%)
Femoral	1 (5%)
Robotically treated lesions per patients	
One lesion	17 (81%)
Two lesions	4 (19%)
Patients with manual treatment of additional lesion(s)	1 (5%)
Lesion/procedural characteristics on lesion level (*N* = 25 lesions)	
Target vessel	
Left anterior descending artery	13 (52%)
Left circumflex artery	6 (24%)
Right coronary artery	6 (24%)
Lesion complexity (ACC/AHA classification)	
Type A or B1	7 (28%)
Type B2 or C	18 (72%)
CTO	0 (0%)
Ostial lesion	0 (0%)
Bifurcation lesions	6 (24%)
With side-branch fenestration	4 (16%)
With side-branch stenting	2 (8%)
Left main	0 (0%)
In-stent restenosis	1 (4%)
Lesion length, mm	25.0 [10.0]
Reference vessel diameter, mm	2.8 [0.5]
% diameter stenosis, %	80% [10%]
Wire type	
Standard	24 (95%)
Hydrophilic/CTO with robot	0 (0%)
Hydrophilic/CTO after manual conversion	1 (4%)
Automatic robot movements applied	23 (92%)
Manual recanalization prior to robot activation	0 (0%)
Lesion predilation	22 (88%)
Number stents or DCB^a^ per lesion	
1	17 (68%)
2	6 (24%)
3	2 (8%)
Mean stent diameter, mm	2.8 [0.5]
Total stent length, mm	30.0 [10.5]
Postdilation	16 (64%)
Procedure duration, radiation and contrast volume on procedure level (*N* = 21)	
Procedure time, min	47 [29]
Robotic time, min	37 [34]
Fluoroscopy time, min	11.5 [10.5]
Dose-area-product, cGy^a^cm^2^	5,118 [3,768]
Contrast media, ml	194 ± 64

Values are mean ± SD, median [interquartile range], or count (%). CTO, chronic total occlusion; DCB, drug coated balloon.

^a^ 1 lesion was treated with DCB.

**Figure 2:**
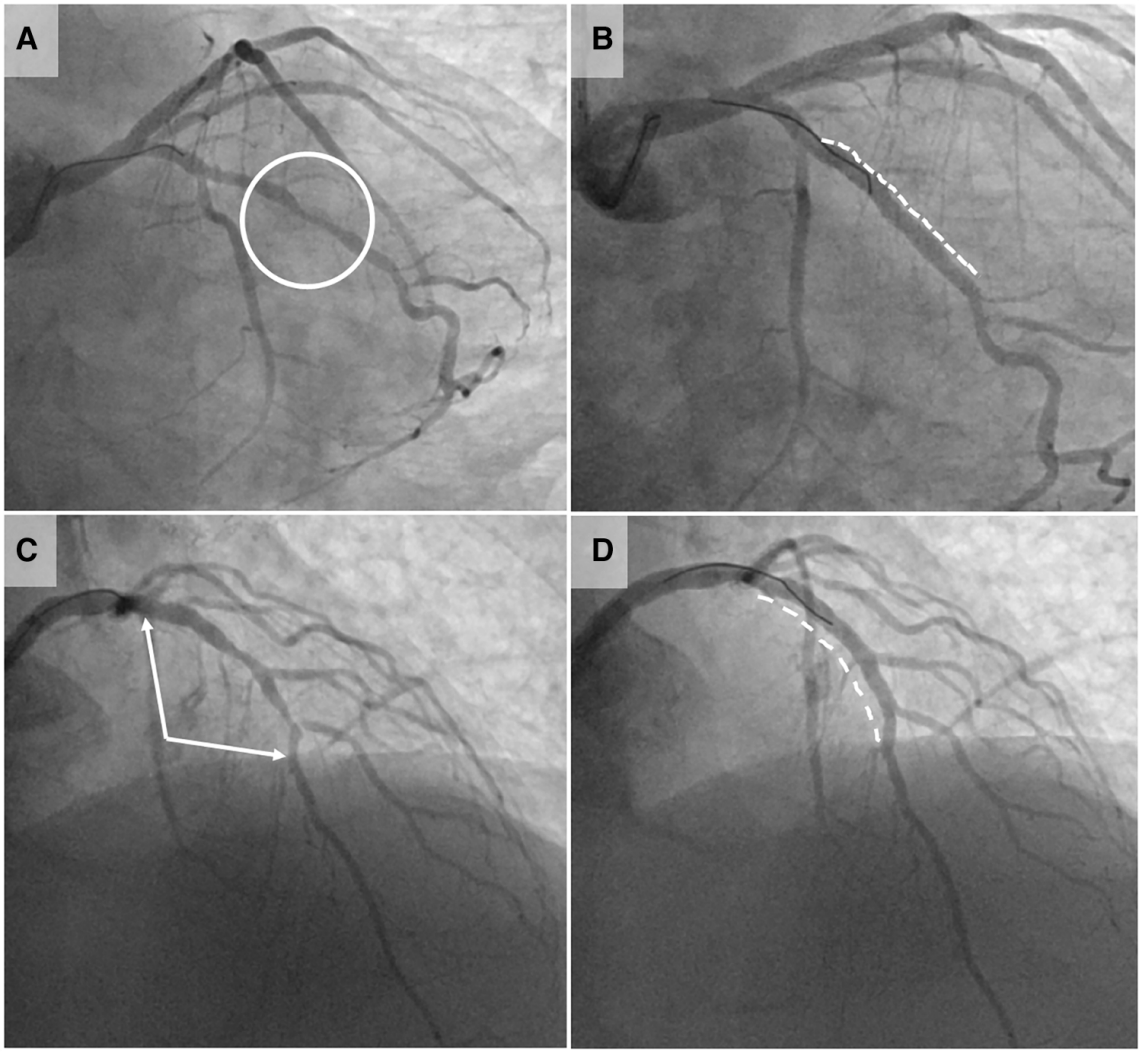
Case illustrations of robotic-assisted PCI. Pre- and post-interventional coronary angiography images of two exemplary lesions treated by robotic-assisted percutaneous coronary intervention (RA-PCI). Panels (**A**) (pre-interventional) and (**B**) (post-interventional result) illustrate the first case conducted robotically at our institution: a focal lesion of the first marginal branch (circle in panel **A**). After manual intubation of the left main with a guiding catheter, the robotic system was connected. The procedure was thereafter conducted robotically by the operator in the cockpit, whereas coronary device exchanges were done by the Catheterization Laboratory technician. The lesion was treated with predilation, implantation of one stent (dashed line in panel **B**) and postdilation. This simple first case pointed to one challenge faced by the Catheterization Laboratory technicians. The coronary devices need to be exchanged manually after opening the single-use cassette. The implemented fixation gadget on the cassette does not provide sufficient guidewire fixation to guarantee wire immobilization during device exchange process, allowing the wire to be pulled or pushed during manipulation. Besides extensive training of the Catheterization Laboratory technicians, the selection of straightforward lesions with low risk for predilation-related dissections is important during the implementation phase of RA-PCI. Panel (**C**) (pre-interventional) and (**D**) (post-interventional result) show one of the latest cases (74 year-old patient) conducted completely robotically. The lesion involved the proximal and mid left anterior descending artery (LAD) including the bifurcation of the second diagonal branch (between arrows in panel **C**). This bifurcation lesion required the use of two coronary guidewires, which necessitated the technician to switch the wires between drive lane and “parking” position several times. Two stents were implanted in the LAD (dashed line in panel **D**) and the diagonal branch was re-wired and fenestrated all robotically.

All procedures were done by a single operator (JH), assisted by four dedicated Catheterization Laboratory technician (range between 2 and 9 procedures per assistant). In 4 patients, two lesions were treated robotically during the same procedure, and one other patient underwent manual treatment of an additional lesion (not planned to be treated robotically). The majority of robotically treated lesions (72%) were type B2 or C, with 24% bifurcation lesions requiring side branch intervention. Figure 2 shows an example of two lesions undergoing robotic PCI. Median procedure time was 47 min [interquartile range: 29 min], robotic time 37 [34] min, and fluoroscopy time 11.5 [10.5] min. Median dose-area-product was 5,118 cGy*cm^2^ [3,768] and mean contrast volume used was 194 ± 64 ml.

### Procedural outcomes of robotic-assisted PCI

**Figure 3:**
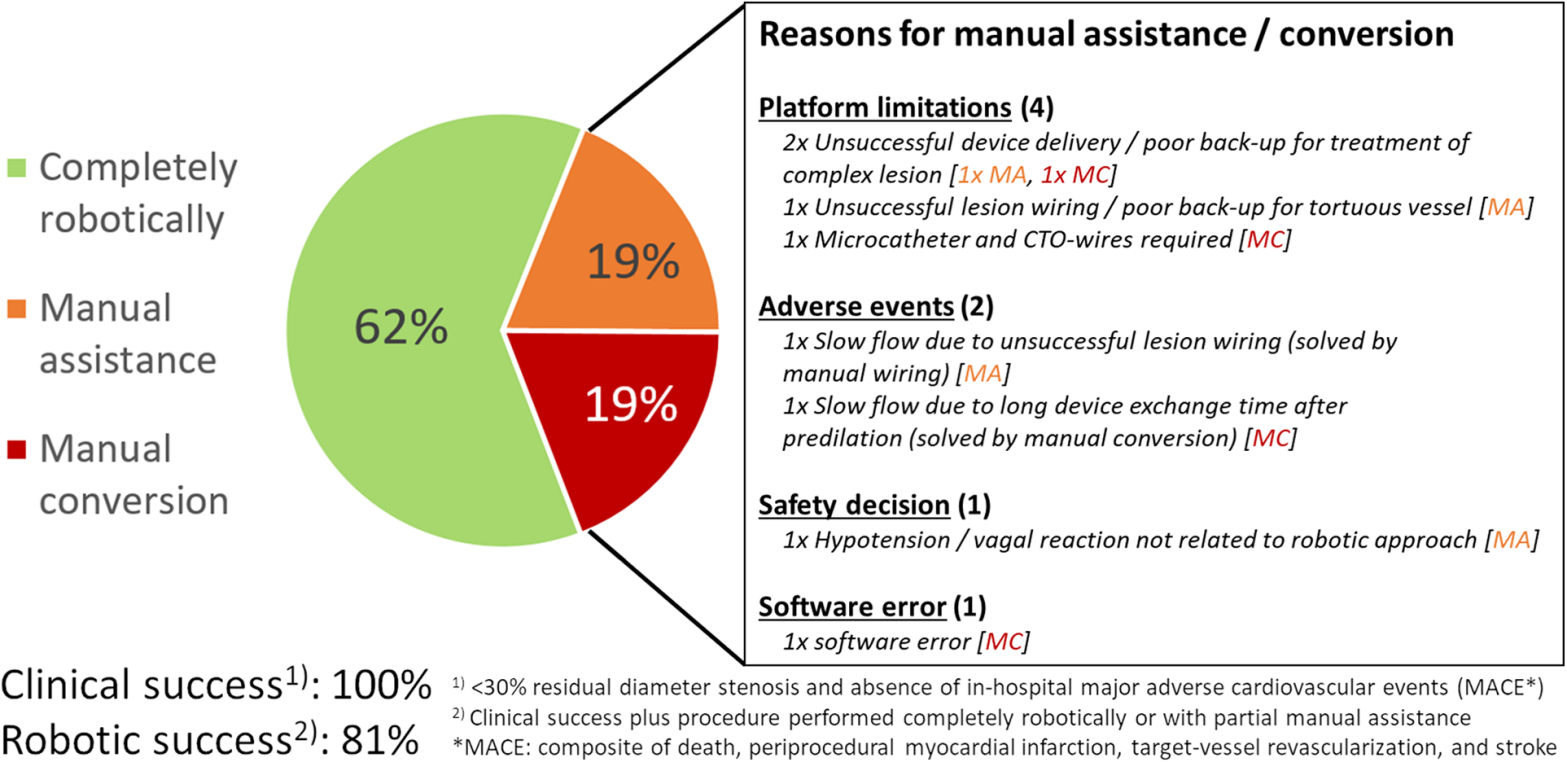
Peri-procedural outcome of robotic-assisted PCI. The pie chart on the left side shows the percentage of cases treated completely robotically (green), with manual assistance (orange) and with manual conversion (red). Reasons for manual assistance [MA] and manual conversion [MC] are summarized on the right side. MA, manual assistance; MACE, major adverse cardiovascular events; MC, manual conversion.

Procedural outcomes are summarized in Table 3. Clinical success was achieved in all cases and robotic success in 81% of procedures (62% completely robotic-guided and 19% with partial manual assistance). Manual conversion was required during 4 procedures (19%). Reasons for manual assistance or conversion are summarized in Figure 3. Manual conversion was needed due to platform limitations or poor guiding catheter support in two cases (characterized by unsuccessful robotic device delivery or the need for use of a microcatheter), a transient adverse event in one case (angiographic slow flow due to prolonged device exchange time after predilation, which was resolved by manual conversion), and a software error in one case. Similarly, most frequent reason for manual assistance were platform limitations or poor guiding catheter support in two cases (characterized by unsuccessful robotic lesion wiring or device delivery), a transient adverse event in one case (angiographic slow flow due to unsuccessful lesion robotic lesion wiring, which was resolved by manual wiring), and a safety consideration in one case during an episode of hypotension/vasovagal reaction not related to the robotic approach.

**Table 3:** In-hospital and one-year clinical outcomes.

	*N* = 21 patients/procedures
**Clinical success**	**21** (**100%)**
**Robotic success**	**17** **(81%)**
Completely robotically	13 (62%)
Manual assistance	4 (19%)
**Manual conversion**	**4** **(19%)**
Peak cardiac enzymes and clinical in-hospital follow-up	
Peak creatine kinase, U/L	108 [114]
Peak TnT-hs, µmol/L	16 [34]
In-hospital MACE^a^	0 (0%)
Antithrombotic Therapy at discharge	
Acetylic salicylic acid	21 (100%)
Clopidogrel	7 (33%)
Prasugrel	3 (14%)
Ticagrelor	11 (52%)
Direct oral anticoagulant	1 (5%)
One-year clinical outcomes	
Target lesion failure^b^	0 (0%)
All-cause death	0 (0%)
Myocardial infarction	1 (5%)
Target-vessel myocardial infarction	0 (0%)
Non-target-vessel myocardial infarction	1 (5%)^c^
Repeat revascularization	1 (5%)
Target-lesion revascularization	0 (0%)
Non-target-lesion revascularization	1 (5%)^c^
Stroke	0 (0%)

Values are mean ± SD, median [interquartile range], or count/events (%).

MACE, major acute cardiovascular events; TnT-hs, high-sensitivity Troponin T.

^a^ MACE was defined as the composite of death, (peri-procedural) myocardial infarction, target-vessel revascularization, and stroke.

^b^ Target lesion failure is defined as the composite of cardiac or unclear death, target-vessel myocardial infarction, or target lesion revascularization.

^c^ One patient suffered non-target-vessel myocardial infarction and underwent revascularization of an in-stent restenosis in a non-robotically treated lesion (four months after robotic procedure).

### In-hospital and long-term clinical outcomes

In-hospital peak cardiac enzymes and discharge medication as well as long-term clinical outcomes are summarized in Table 3. No in-hospital MACE occurred. During 1-year follow-up no target lesion failure had occurred. One patient suffered a non-target-vessel myocardial infarction not related to the index PCI requiring repeat PCI of another lesion four months after RA-PCI.

## Discussion

A RA-PCI program was initiated at Bern University Hospital in June 2021 and 25 lesions in 21 patients were safely performed during the first seven months without clinically relevant robot-associated complications. All procedures conducted in the early implementation phase of RA-PCI were clinically successful, and 81% of the interventions were performed either completely robotic-guided or with partial manual assistance. To the best of our knowledge, Bern is the only center in Switzerland, which has used the CorPath GRX system.

### Safety and efficacy of RA-PCI

High rates of clinical success (>95%–100%) are consistently reported in most RA-PCI registries for first- (5, 6) and second-generation robotic CorPath systems (7, 13), across different lesion complexities (12). Two recent studies reported lower clinical success rates, due to higher rates (6.7%) of residual percent diameter stenosis in 30 RA-PCIs conducted in Japan (11) or higher rates of in-hospital MACE rates (7%) in 71 RA-PCIs (with a high proportion of complex lesions including CTOs) conducted in Germany (14).

In our registry, 62% of RA-PCI were done completely robotic-guided and 19% with partial manual assistance. Accordingly, 81% of the procedures were completed robotically with high clinical success and absence of in-hospital MACE (=robotic success). The rate of robotic success in our experiences was lower than that reported in other registries [robotic success of 91.7% in 108 patients in the CORA-PCI registry (6); 85.7% in 112 lesions reported by Lemos et al. (13); or 94.2% in 84 lesions treated in Hamburg, Germany (14)], although the latter two reported robotic success based on lesion rather than procedure level and did not require absence of in-hospital MACE. To some extent, the lower robotic success rate in the present study may be attributable to a lower threshold for manual assistance or conversion to keep the procedure safe and avoid unnecessary delay to complete the intervention which is reflected in the high clinical success rate.

Reasons for manual conversion (in 19% of our cases) were similar as for the cases requiring partial manual assistance. Half of the of manual inputs were related to platform limitations or insufficient back-up (poor guiding catheter or wire support, use of devices not supported by the robotic system), 25% related to transient angiographic slow flow which resolved following manual assistance or conversion, and the remaining reasons were safety measures in a patient with vasovagal reaction and a software error. Similarly, in the CORA-PCI registry, guiding catheter or wiring support issues and robotic platform limitations were the leading causes (85%) for manual assistance or conversion and adverse events accounted for 15% (15). Rare cassette or system errors were also reported from a Japanese registry (16). Predictors for the need of manual inputs in other studies were lesion complexity and higher-graded pre-PCI stenosis (12, 15). This matches our experience and can be explained by the more frequent use of specialty devices, stronger guiding catheter back-up needed for lesion wiring and device delivery, and higher risk for (transient) no-flow due to unsuccessful lesion wiring or longer device exchange times during treatment of such lesions.

No events related to the robotically treated coronary lesion occurred during 1-year follow-up, attesting to the efficacy of the procedure in well selected staged PCI patients. Only one non-target vessel myocardial infarction occurred, which was unrelated to the index procedure. To date, one-year clinical outcome of RA-PCI was only reported from the CORA-PCI registry with a similar rate of MACE (7.8%) (17).

### Experiences during the implementation process

RA-PCI changes the workflow for operators and Catheterization Laboratory technicians. Instead of manual positioning of guidewires, coronary devices, and guiding catheters guided by both visual and tactile feedback, the operator using RA-PCI uses joysticks and relies on the visual feedback of fluoroscopy images and technical safety features of the robotic system. Notwithstanding, RA-PCI does not change the overall strategic approach to coronary intervention, as most standard wires and balloon/coronary stents can be used. Although the system does not support other devices such as microcatheters, intracoronary imaging catheters, and rotational ablation devices, these can still be used manually, hence not limiting the diagnostic and therapeutic armamentarium during PCI. Since the system use is very intuitive, the operator can quickly adapt to the new technology with the benefit of reduced radiation exposure avoiding the need to wear radiation protection gear.

RA-PCI has a major impact on the workflow of Catheterization Laboratory technicians as they need to get adapted to load the robotic system with guidewires and to exchange coronary device-catheters. Although the robotic cassette features a fixation system for the guidewire, the first case (Figure 2) illustrates that guidewire movement during device exchange represents a limitation. Aside from specific training of Catheterization Laboratory technicians in terms of wire handling during device exchange, it is advisable to initiate a RA-PCI program focusing on the intervention of simple lesions. Furthermore, device exchange times are longer than during manual PCI due to additional steps needed (opening of the cassette, removal of the guidewire and the used device from their respective drive lanes and replacing the guidewire and the new device in their lanes followed by closure of the cassette), and the unusually high level of manipulation of the guidewires and devices due to the fix position of the robotic arm. We were unable to observe the expected reduction in manpower needed for RA-PCI. Although the primary operator mainly acts from the cockpit, a tableside assistant familiar with the robotic system is needed. Moreover, an additional cathlab nurse is needed to supply the tableside assistant with PCI material, to administer medication to the patient and to document the peri-procedural course.

### Current limitations of RA-PCI

The procedure time was prolonged by approximately 10 min due to set-up of the robotic system in our study, which is comparable to other reports (6, 8). Mahmud et al. reported that this applies mostly to low complexity procedures, whereas procedure times were similar for RA-PCI and conventional PCI in more complex interventions (6).

The major impact of RA-PCI is the markedly lower radiation exposure of the operator. Increases in procedural case volume by operator, case complexity and radial access by default led to higher operator radiation exposure in the last decades (18, 19), which is associated with a lifetime attributable risk of cancer (19) as well as orthopedic injuries due to the more extended periods in lead aprons (18). RA-PCI studies consistently show >90% (to 99%) reduction in radiation to the operator's head (3, 5, 11) by use of this novel technology. However, since the second operator or the Catheterization Laboratory technician is no more “protected” by the primary operator, measures to minimize radiation exposure to the assistants are of utmost importance. These measures include minimization of fluoroscopy, while the tableside assistant loads the robotic arm, and to ensure that the assistant steps away, when not handling the robotic arm. Furthermore, additional lead-shields may be placed at the position, where the primary operator would usually be positioned. Whether or not radiation exposure to the tableside assistant is higher during RA-PCI remains unclear. One study did not show a significant increase in radiation to the assistant compared with manual PCI (11). While radiation to the patient was similar between 108 RA-PCI compared to 226 manual PCIs in the CORA-PCI registry (6), the largest propensity score matched study to date (registry with 310 RA-PC and 686 manual PCI) reported RA-PCI to be associated with a decrease in radiation exposure to the patient despite similar fluoroscopy times (8). Use of semi-automated wire movements and, in the future direct interaction between the robotic and imaging system or augmented reality systems may further shorten procedure time and radiation dose to exposed persons (20).

Current robotic systems do not support most intracoronary imaging devices, which therefore must be operated manually (12, 16). Aiming for completely robotic procedures should not be a reason to refrain from use of intracoronary imaging, which will be increasingly used in routine clinical PCI practice. Aside from intracoronary imaging catheters, additional devices are not compatible with the current robotic platform. Guide catheter extensions, which may be required to overcome limited guiding catheter support (15, 16, 21), microcatheters and rotational ablation devices must be used manually. However, a newer robotic cassette, inspired by the neurovascular CorPath system, features an adaptor to be connected to a microcatheter broadening the spectrum of lesions potentially eligible to RA-PCI. Another limitation is that only one guidewire and one coronary device (balloon or stent catheter) can be controlled actively at once. In case of bifurcation/trifurcation treatment requiring multiple wires and/or coronary devices, the Catheterization Laboratory technician has to open the cassette and switch the wire/device from the “parking” position to the active drive lane to allow the operator sequential control of multiple wires/devices. However, if simultaneous movement of devices is necessary (e.g., for kissing-ballooning), manual input is likely required. Future systems should feature more than a single active wire and device lane.

In several surgical fields, utilization of robotic systems are widely implemented due to advantages in terms of ergonomics and decreased fatigue, higher precision and enhanced visualization, although, evidence of clinically relevant patient-oriented benefits is limited (22). While use of robotics is approaching 30% in some general and urologic surgery series (1), robotics in PCI is still in its infancy.

The slow adoption of RA-PCI systems in Switzerland and worldwide despite consistently reported favorable safety and reduction in radiation exposure to the operator may be related to the additional costs (23) not covered by the healthcare systems, need for highly qualified tableside assistants, lack of clinical benefit and limitation in use of more specialized PCI equipment. The possibility of long-distance remote PCI (“tele-stenting”), which has already been performed in India (24), is unlikely to become relevant in Switzerland given the high density of catheterization laboratories, but may be an interesting opportunity for large countries (25). Nevertheless, the slow adoption resulted in the recent retraction of the robotic CorPath GRX system by the manufacturer due to economic considerations although competitive products continue to be commercialized.

### Limitations

This study has some limitations. Although baseline, procedural and clinical outcome data were collected prospectively, the design of this investigation was a retrospective cohort study. To avoid selective reporting bias, we used the most commonly applied definitions for the two endpoints of interest. In addition, the study population was small and consisted of highly selected patients with known coronary anatomy prior to RA-PCI. Therefore, the observed outcomes may not apply to more complex and ad-hoc procedures. All procedures were conducted by a single operator and four dedicated Catheterization Laboratory technicians. This resulted in a short learning curve for the robotic team. However, it does not allow to examine interoperator variability. Moreover, this study did not allow to investigate reduction in radiation exposure to the operator, since operator dosimetry was not available on a procedure-level.

## Conclusions

RA-PCI can safely be performed without clinically relevant robot-associated complications in the early implementation phase of PCI robotic program with approximately 80% of procedures conducted completely robotically or with partial manual assistance. To allow more efficient RA-PCI and broader adoption, future iterations of the robotic platform should allow for handling of multiple wires and coronary devices simultaneously, provide autonomous loading of devices, integrate intracoronary imaging options and direct interaction with the imaging system.

## Data Availability

The raw data underlying this article will be shared upon reasonable request to the corresponding author.

## Appendix A: Analysis of robotic success and individual robotic steps on lesion level

Part 1: Definitions of robotic steps

Percentage of successful robotic steps were calculated for guidewire delivery (defined as successful robotic lesion wiring divided by the number of lesions in which robotic wiring was attempted), delivery of all predilation balloons (defined as successful robotic balloon delivery divided by the number of lesions in which robotic balloon delivery was attempted), and delivery of stent and postdilation balloon, (both defined analogously, respectively) and device retrieval. The latter was defined as the percentage of successful robotic retrieval of all devices divided by the number of lesions in which any robotic device retrieval was attempted, e.g. excluding lesions in which manual conversion occurred prior to any robotic device retrieval attempt.

Part 2: Robotic success on lesion level and success rates of robotic steps

**Table:**

Robotic success on lesion level (*n* = 25)	
Angiographic lesion success, *n* (%)	25 (100%)
Robotic lesion success, *n* (%)	17 (68%)
Manual assistance, *n* (%)	4 (16%)
Manual conversion, *n* (%)	4 (16%)
Success rates of individual robotic steps	
Robotic delivery of guidewire, successful/attempted (%)	23/25 (92%)
Robotic delivery of predilation balloon, successful/attempted (%)	18/20 (90%)
Robotic delivery of stent, successful/attempted (%)	20/23 (87%)
Robotic delivery of postdilation balloon, successful/attempted (%)	12/13 (92%)
All devices retrieved successfully, successful/attempted (%)	22/23 (96%)

The analysis of individual robotic steps showed, that robotic guidewire delivery was successful in 92% (23/25 attempts), robotic delivery of predilation balloon in 90% (18/20), stent delivery in 87% (20/23), and delivery of postdilation balloon in 92% (12/13). Robotic retrieval of all devices was successful whenever attempted, except in one case, where manual assistance was needed due to guiding catheter extubation during an unsuccessful robotic retrieval attempt of a non-advancable stent in a severely calcified lesion.
